# Consensus Guidelines for the Prevention and Management of Periprocedural Complications of Transcatheter Patent Ductus Arteriosus Closure with the Amplatzer Piccolo Occluder in Extremely Low Birth Weight Infants

**DOI:** 10.1007/s00246-021-02665-3

**Published:** 2021-06-30

**Authors:** Shyam Sathanandam, Dan Gutfinger, Brian Morray, Darren Berman, Matthew Gillespie, Thomas Forbes, Jason N. Johnson, Ruchira Garg, Sophie Malekzadeh-Milani, Alain Fraisse, Osman Baspinar, Evan M. Zahn

**Affiliations:** 1 LeBonheur Children’s Hospital, University of Tennessee, 848 Adams Avenue, Memphis, TN 38103 USA; 2 Abbott Structural Heart, Sylmar, CA USA; 3 Seattle Children’s Hospital, Seattle, WA USA; 4 Nationwide Children’s Hospital, Columbus, OH USA; 5 Children’s Hospital of Philadelphia, Philadelphia, PA USA; 6 Children’s Hospital of Michigan, Detroit, MI USA; 7 Cedars-Sinai Medical Center, Los Angeles, CA USA; 8 Necker University Hospital for Sick Children, Paris, France; 9 Royal Brompton Hospital, London, UK; 10 Kayseri City Training and Research Hospital, Kayseri, Turkey

**Keywords:** Transcatheter PDA closure, Amplatzer Piccolo Occluder, Device embolization, Device migration, Device protrusion, Aortic obstruction, Pulmonary artery obstruction, Tricuspid regurgitation, Cardiovascular injury

## Abstract

Transcatheter closure of patent ductus arteriosus (PDA) in premature infants is a feasible, safe, and an effective alternative to surgical ligation and may be performed with an implant success rate of 97%. Major procedural complications related to transcatheter PDA closure in extremely low birth weight (ELBW) infants are relatively infrequent (< 3%) ,but may be associated with a fatality if not optimally managed. Operators performing transcatheter PDA closures should be knowledgeable about these potential complications and management options. Prompt recognition and treatment are often necessary to avoid serious consequences. With strict guidelines on operator training, proctoring requirements, and technical refinements, transcatheter PDA closure in ELBW infants can be performed safely with low complication rates. This article summarizes the consensus guidelines put forward by a panel of physicians for the prevention and management of periprocedural complications of transcatheter PDA closure with the Amplatzer Piccolo Occluder in ELBW infants.

## Introduction

In recent years, there has been a growing interest in utilizing transcatheter patent ductus arteriosus (PDA) closure in extremely low birth weight (ELBW) infants as a less invasive alternative to surgical ligation and a more effective treatment option compared to medical therapy [1–3]. With increased procedural experience a modified technique for transcatheter PDA closure was developed to minimize adverse events in ELBW infants [4–6]. The modified implant technique avoids arterial access and exclusively utilizes a transvenous antegrade approach guided by fluoroscopy, venous angiography, and transthoracic echocardiography (TTE) with placement of the entire device within an intraductal position to avoid aortic and pulmonary artery protrusion [7, 8].

The Amplatzer Piccolo™ Occluder (Abbott Structural Heart, Plymouth, MN, USA) was designed for use in premature infants [9] and is the first device approved for transcatheter PDA closure in infants as small as 700 grams [10]. In the United States, a single arm, prospective, multicenter, non-randomized study was conducted to evaluate the Amplatzer Piccolo Occluder to treat PDA in patients ≥ 700 g, that yielded an implant success rate of 95.5% (191/200) overall and 99% in patients ≤ 2 kg (99/100). Although the incidence of periprocedural complications is relatively low, there is opportunity to reduce the event rates further by defining guidelines for the prevention and management of adverse events. In 2020 a panel of interventional congenital cardiologists, echocardiography imaging cardiologists, and representatives from the device manufacturer convened to review and analyze a series of transcatheter PDA closure cases performed in ELBW infants using the Amplatzer Piccolo Occluder where key adverse events occurred. Based on this analysis insights into the mechanism of these adverse events were gained and guidelines for the prevention and management of these complications were developed by the panel. This article presents the incidence, mechanism, and consensus guidelines for the prevention and management of key periprocedural complications as defined in Table 1.

**Table 1:** Key Periprocedural Complications of Transcatheter PDA Closure in Premature Infants

Complication	Definition
Device Embolization	The entire device travels from the ductus arteriosus to another intravascular location. Device embolization is more common to the pulmonary artery than the aorta and can frequently be retrieved with a vascular snare
Device Migration	The device moves from the original implant location within the ductus and partially protrudes outside of the ductus, while the rest of the device remains within the duct. Migration might occur due to ductal vasoconstriction and shortening causing the device to be pushed partially out of the duct
Device Protrusion	A portion of the device following release projects into either the aorta or the LPA. The degree of protrusion can range from clinically insignificant to causing severe aortic obstruction or LPA obstruction. Device protrusion usually occurs as a result of non-optimal device position, over-sizing, or migration
Tricuspid Valve Regurgitation	Incompetence of the tricuspid valve resulting in leakage of blood from the right ventricle into the right atrium during right ventricular contraction. Tricuspid valve regurgitation may occur as a result of injury to the valve leaflets or chordae during passage of guidewires and catheters across the valve
Cardiovascular Injury	Injury to the vasculature or heart that may range anywhere from partial thickness vessel wall injury to a full thickness vessel wall injury or perforation with bleeding into the vessel wall (dissection) or into a free space outside the vessel or heart resulting in a hematoma, pericardial effusion or a cardiac tamponade
Residual Shunt	Incomplete closure of the PDA that results in persistent flow across the PDA that may occur around or through the device
Hemolysis	The destruction of red blood cells most commonly due to high shear stress commonly caused by high flow through a narrow residual shunt channel
Contrast Induced Nephropathy	Impairment of renal function that occurs within 24 to 72 h of intravenous contrast administration

## Device Embolization

Device embolization is a known complication of transcatheter PDA closure but is of particular concern in ELBW infants as surgical retrieval may be technically challenging and associated with a high degree of morbidity due to patient size. [4, 11, 12]. Most instances of device embolization occur either during the procedure or immediately post procedure into one of the pulmonary artery branches and can be safely retrieved via a transcatheter approach. Aortic embolization is much less common than embolization into the pulmonary artery.

A distinction is made between device embolization and migration as the management of these two complications may be different. Device embolization occurs whenever the entire device travels from the ductus arteriosus to another intravascular location. Device migration occurs whenever the device moves from the original implant location within the ductus and partially protrudes into surrounding structures while still partially remaining within the duct (Table 1). While device retrieval is recommended for cases of embolization, there may be cases of migration in which device retrieval may not be necessary. Therefore, device migration will be discussed later in the context of device protrusion and residual shunt.

### Incidence, Mechanism, and Clinical Presentation

The overall incidence of device embolization with the Piccolo Occluder based on the premarket trial conducted in the United States (ClinicalTrials.gov identifier: NCT03055858) was 2.5%. In the five cases during this trial, device embolization occurred intraprocedurally into a pulmonary artery branch within minutes following device release [10]. The embolization rate among infants ≤ 2 kg was 2%, while the embolization rate among children > 2 kg was slightly higher at 3%. From other studies of the Piccolo occluder, the reported incidence of device embolization in infants ≤ 2 kg (Table 2) was 3.1% (range 0 to 8.3%) with all devices embolizing into the pulmonary artery branches [13–18]. Embolization is more common into the left pulmonary artery (~ 65%) but can happen into the right pulmonary artery (~ 30%) or the main pulmonary artery (~ 5%) as well. It most commonly occurs during the procedure shortly after the device is released or within 24 h after the procedure. Embolization detected beyond 24 h may represent cases where the device embolized earlier but was not recognized due to absence of symptoms and limited post-procedural imaging.

**Table 2:** Complications reported for transcatheter PDA closure in premature infants ≤ 2 kg using the Amplatzer Piccolo Occluder

Author & Year	Weight at Implant (g)	Implant Success (%)	Embolization (%)	Aortic Obstruction (%)	LPA Obstruction (%)	Tricuspid Regurgitation (%)	Cardiovascular Injury (%)	Procedure-related mortality (%)
Baspinar 2015 (N = 12) [13]	1538 ± 239 (1180–2000)	10/12 (83%)	1 (8.3%)	0 (0.0%)	0 (0.0%)	0 (0.0%)	1 (8.3%)	1 (8.3%)
Rodriguez 2017 (N = 27) [14]	1325 ± 281 (1000–1980)	27/27 (100%)	2 (7.4%)	0 (0.0%)	0 (0.0%)	0 (0.0%)	0 (0.0%)	0 (0.0%)
Moreville 2017 (N = 25) [15]	1133 ± 302 (680–1700)	24/25 (96%)	0 (0.0%)	0 (0.0%)	1 (4.0%)	0 (0.0%)	1 (4.0%)	1 (4%)
Pamukcu 2018 (N = 26) [16]	1396 ± 433 (750–2000)	22/26 (85%)	2 (7.7%)	1 (3.8%)	0 (0.0%)	0 (0.0%)	1 (3.8%)	0 (0.0%)
Milani 2019 (N = 73) [17]	≤ 2000	73/73 (100%)	0 (0.0%)	0 (0.0%)	3 (4.1%)	3 (4.1%)	0 (0.0%)	0 (0.0%)
Regan 2020 (N = 64) [18]	1200 (1025–1700)	63/64 (98%)	2 (3.1%)	1 (1.6%)	0 (0.0%)	2 (3.1%)	0 (0.0%)	0 (0.0%)
Sathanandam 2020 (N = 100) [10]	1248 ± 348 (700–2000)	99/100 (99%)	2 (2.0%)	2 (2.0%)	0 (0.0%)	5 (5.0%)	0 (0.0%)	0 (0.0%)
All	**1282 ± 353 (680–2000)**	**318/327 (97%)**	**9 (2.8%)**	**4 (1.2%)**	**4 (1.2%)**	**10 (3.1%)**	**3 (0.9%)**	**2 (0.6%)**

Device sizing is to be performed according to the two sizing tables for children ≤ 2 kg and > 2 kg, respectively, provided by the device manufacturer in the instructions for use (IFU) [19]. In infants ≤ 2 kg the device sizing table specifies placement of the entire device with both retention discs within the duct (*intraductal placement*) and ensures that the diameter of the retention discs is at least 50% larger than the minimal ductal diameter. In children > 2 kg the device sizing table specifies placement of the central waist across the entire length of the duct with the retention discs placed just outside the duct or within the ampulla (*extraductal disc placement*) and ensures that the diameter of the central waist is at least 50% larger than the minimal ductal diameter. A *proper device position* is defined as intraductal in infants ≤ 2 kg without causing aortic or LPA obstruction, and as an extraductal disc placement in children > 2 kg also without causing aortic or LPA obstruction. A *proper device orientation* is defined as being coaxially aligned with the long axis of the ductus and pointing toward 10 o’clock on a 90° lateral fluoroscopy view.

Table 3 outlines potential mechanisms for device embolization. The most common causes for device embolization are improper device positioning, or selection of a device size that has a diameter that is smaller than the minimal ductal diameter. This may occur whenever the ductal anatomy and dimensions are not well visualized on imaging studies or in the presence of ductal spasm which results in an underestimation of the ductal diameter [20, 21]. Iatrogenic dislodgement of the device can occur due to an inadvertent push of the device with a wire or catheter following release, or inadvertent, slow pull on the device with the delivery wire due to delayed release of the device. Embolization may also occur as a result of inadvertent unscrewing of the device from the delivery wire during attempts to position the device. A sudden increase in blood flow or intrathoracic pressure in the early post-implant period can also result in embolization. Lastly, infants > 2 kg with a vasoactive ductus, high blood flow, and a conical PDA morphology may be at a higher risk for device embolization compared to smaller infants with a tubular ductus.

**Table 3:** Mechanisms for Device Embolization

Mechanisms for device embolization	
Inadequate Imaging	Underestimation of ductal dimension due to incomplete visualization of the ductus
Device Size	Implanted device is too small for the encountered anatomy
Ductal Spasm	Instrumentation of the ductus causes smooth muscle constriction leading to underestimation of ductal diameter
Device Malposition	Intraductal disc placement in larger infants (> 2 kg) Incorrect device orientation or shape
Delivery System	Anterior tension on the device by delivery wire. Delivery catheter preventing device to stay co-axial along the length of the duct
Operator related	Pushing delivery wire or catheter forward after device release. Prolonged time interval between device placement and release. Inadvertent unscrewing of the device from the delivery wire. Unfamiliarity with device sizing and placement guidelines
Patient related	Vigorous activity resulting in sudden increase in blood flow or intrathoracic pressure
Duct Morphology	Implanted device shape does not match shape of ductus

To the best of our knowledge device embolization into a pulmonary artery branch is well tolerated by most infants > 1 kg, and there are no known reports of any child developing acute hemodynamic instability due to a device embolization. If device embolization occurs after discharge from the catheterization laboratory, the clinical presentation consists of a return of a continuous murmur associated with shunting across the PDA on TTE and an X-ray image confirming the device in a location other than the PDA. Typically, there may not be any acute symptoms as in nearly all cases the device embolizes into the pulmonary circulation without fully obstructing pulmonary blood flow. The presence of arrhythmias, ischemic changes on an electrocardiogram (EKG), oxygen desaturation, or limb ischemia are less commonly seen with embolization of the Piccolo Occluder. However, infants < 1 kg could potentially develop acute systemic arterial desaturation if the device embolized to the pulmonary circulation, or signs of systemic hypoperfusion to the gut, kidneys or the lower extremities, if embolized into the aorta. Therefore, post-procedure monitoring and assessment of device position during the first 24 h post implant is critical so that cases of device embolization may be identified and treated promptly.

### Prevention

Device embolization is best prevented by ensuring that the device size selected is based on accurate PDA measurements derived from a combination of high-quality TTE and angiographic images coupled with a clear understanding of the unique sizing characteristics of this device. TTE measurements of the ductal dimension should be obtained prior to instrumenting the PDA, and angiography should allow for full visualization of the ductal dimensions across the entire length of the ductus throughout the cardiac cycle.

- Accurate assessment of the PDA morphology and dimensions is crucial and will help select a suitable device type and size. Whenever possible use both TTE and angiography to obtain PDA dimensions before and after instrumentation.
- Ensure that the Piccolo occluder is the best device choice for the PDA dimensions and morphology. There are ductal morphologies such as a type A or conical PDA for which the Piccolo occluder may not be the preferred choice.
- A hand injection angiogram performed with a *pullback technique* where contrast is injected beginning in the aortic isthmus and while pulling back the catheter used for injecting contrast from the aortic isthmus to the pulmonary artery may help delineate more fully the PDA morphology and allow for accurate measurement of the PDA dimensions across the entire length of the ductus
- When instrumenting the PDA there is a potential for ductal spasm which may result in underestimation of the PDA diameter [20, 21]. Therefore, it is important to also assess the PDA dimensions using TTE prior to instrumentation. However, TTE may underestimate the PDA length [8].
- If there is inconsistency between angiography and TTE regarding the PDA size, then consider selecting the device size based on the modality that provides the larger PDA size. As the use of a larger device size may result in protrusion into the aorta or the left pulmonary artery (LPA), it is important to ensure that the most reliable imaging modality is utilized for guiding device size selection. Inconsistencies between imaging modalities may be due to multiple factors, such as variation in imaging angulation and windows, amount of contrast injected, and/or ductal vascular tone.
- Device positioning depends on infant weight. In small infants (≤ 2 kg), the Piccolo occluder length is chosen to achieve intraductal placement, whereas in larger infants (> 2 kg), the occluder length is chosen to achieve an extraductal disc placement. The extraductal positioning in larger infants who have higher blood flow provides improved positional stability and minimizes the potential for device embolization.
- Use of a longer device (4 mm rather than the 2 mm waist) in small infants (≤ 2 kg) when the duct is longer than 12 mm may potentially provide a more secure position of the device within the duct and possibly allow for a smaller diameter device which may limit compression on surrounding anatomical structures (i.e., a 4 mm × 4 mm device may be a better choice than a 5 mm × 2 mm device in infants ≤ 2 kg with a large diameter ductus with length). However, for infants < 1 kg, using the shorter, 2 mm length device is preferrable whenever possible (i.e., for the same PDA as above, use the 4 mm × 2 mm device if patient is < 1 kg).
- During device preparation make sure to unlock the occluder from the delivery wire by turning the occluder counterclockwise 1/8 of a turn to make disconnection easier, while ensuring that the occluder remains threaded onto the delivery wire.
- Following device deployment pull back the delivery catheter away from the device so that the floppy section of the delivery wire is exposed to permit imaging assessment of device position and orientation without the delivery system applying superior tension onto the device. This same maneuver should also be performed prior to device release.
- Do not release the occluder from the delivery wire if the position of the occluder is not stable, or if the occluder shape and orientation are not correct.
- A residual shunt may be observed by color Doppler through the center of the device. However, if a residual shunt on the TTE is visualized to go around the device, then either the device has a diameter that is too small for the encountered PDA diameter or there is a malposition of the device. Effort should be made to reposition the device and re-assess for residual shunting. If the residual shunt around the device is still present despite proper orientation of the device, then one should consider using a device that is one size larger.
- Gently release the device and minimize interaction with the delivery catheter. Once the occluder is released do not push the delivery wire or catheter forward since it may hit the device and cause embolization.
- Following the release of the device continue to monitor the infant for several minutes prior to removing the vascular access sheath to ensure the device remains in stable position. If there are concerns, continue to monitor the infant post procedure and perform TTE or fluoroscopy to make sure the occluder is in the correct position before the patient is transferred out of the catheterization laboratory.

### Percutaneous Retrieval of an Embolized PDA Occluder from the Pulmonary Circulation

Despite taking adequate steps to prevent its occurrence, device embolization can happen, and it is important to always be prepared for a possible retrieval [22, 23]. Preparation includes access to a transcatheter snare kit, diagnostic catheters, retrieval sheaths and an onsite surgeon (Table 4). Most infants > 1 kg tolerate having an embolized device within the pulmonary circulation but may become hemodynamically unstable during attempts to retrieve the device secondary to instrumentation pushing on anatomical structures and keeping right heart cardiac valves open. Devices in the pulmonary circulation can nearly always be retrieved (> 95%) with a transcatheter snare, but sometimes challenges may be encountered, and it may be necessary to proceed with surgery to retrieve the device or abandon any further attempts to retrieve the device and postpone retrieval to a future time.

**Table 4:** Device Retrieval Tool Kit

Retrieval Sheaths	4F Cook Flexor Ansel Guiding Sheath with Check-Flo Hemostasis Valve (45 cm; ANL0; 0.018 or 0.035; G48186)* 5F Cook Flexor Ansel Guiding Sheath with Check-Flo Hemostasis Valve (45 cm; ANL0; 0.018 or 0.035; G44153)*
Diagnostic Catheters for accessing LPA	4F Terumo Glidecath (100 cm; Multi-Purpose; 0.038; CG418) 3.3F Pedivascular Mongoose Pediatric (60 cm; JB1; 0.030; A-3JB1-60/0008) 3.3F Pedivascular Mongoose Pediatric (60 cm; JR2; 0.030; A-3JR2-60/0046)
Diagnostic Catheters for accessing RPA	4F Merit Performa Pediatric Judkins Right 2.0 (70 cm; JR 2.0; 0.038, 7701-B0) 4F Merit Performa Pediatric Judkins Right 2.5 (70 cm; JR 2.5; 0.038, 7701-C0) 4F Merit Impress Berenstein Hydrophilic Catheter (65 cm; 0.038; 46538BER-H) 4F Terumo Glidecath (65 cm; Angle; 0.038; CG415) 4F Terumo Glidecath (65 cm; C2; 0.038; CG409) 4F Terumo Glidecath (100 cm; JB1; 0.038; CG405)
Guidewires	0.035 Wholey Wire 0.035 Angled or Straight Glide Wire 0.014 Wire of choice (All-Star, Balanced Middle Weight, or others)
Snares	5 mm, 7 mm, and 10 mm Amplatz Gooseneck snare 3.2F Merit Ensnare (compatible with the 3.3F Mongoose catheters) 5 mm PFM Multi-snare (125 cm; 0.035; 147305V2)

* Suitable for retrieving all sizes of Piccolo occluders

Once an embolized device in the pulmonary circulation is snared it should be retrieved through a long retrieval sheath that has been previously positioned in the main pulmonary artery (MPA) to avoid pulling the device across the pulmonic and tricuspid valves. However, whenever the long retrieval sheath cannot be safely advanced into the MPA it might be simpler as an absolute last resort to gently retrieve the device unguarded across the pulmonic and tricuspid valves despite the potential risk of damaging the valves. This is because multiple attempts to cross the tricuspid valve with a retrieval sheath may alone result in an injury to the valve or other structures.

**Fig. 1:**
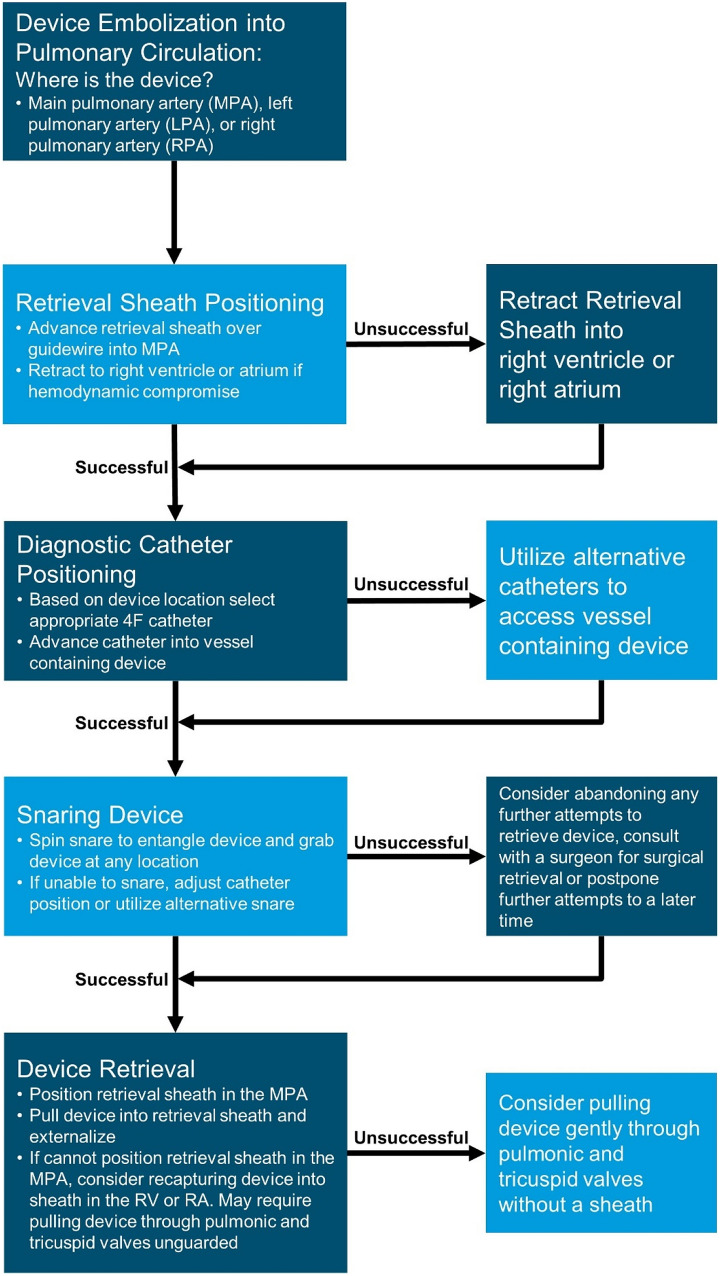
Algorithm to manage device embolization. MPA, main pulmonary artery; LPA, left pulmonary artery; RPA, right pulmonary artery. RV, right ventricle; RA, right atrium

A management algorithm for device embolization into the pulmonary circulation is outlined in Fig. 1 along with the following management considerations.

**Fig. 2:**
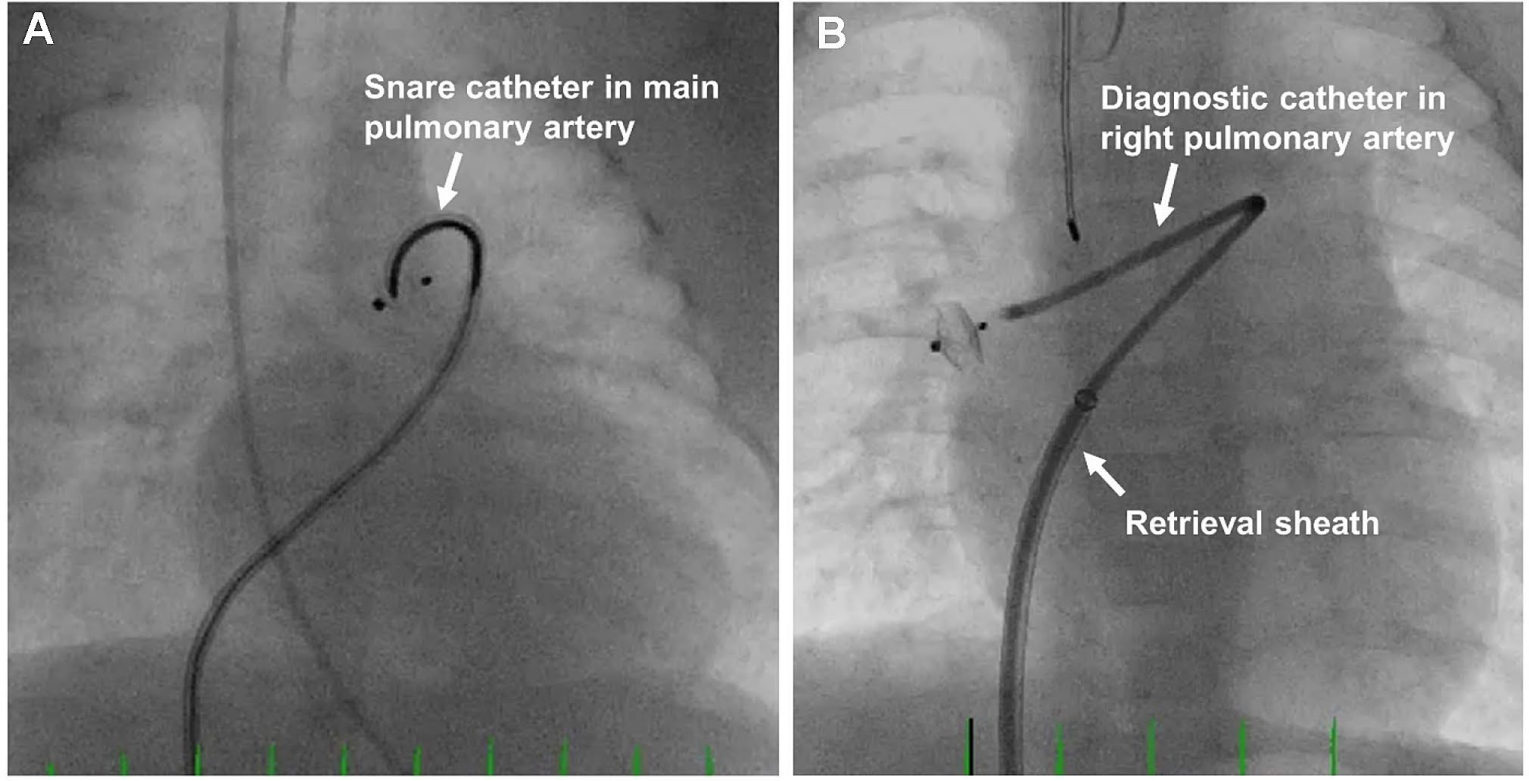
Retrieval of device embolization into the main pulmonary artery (**A**) and right pulmonary artery (**B**)

- Administer heparin to ensure an ACT > 200 s if there is no contraindication for anticoagulation. If not already done, send a type and cross in the event a blood transfusion is needed secondary to blood loss from multiple catheter exchanges.
- Select a suitable retrieval sheath that is large enough to retrieve the embolized device (Table 4). A 4F or 5F Cook Flexor Ansel guiding sheath may be used to retrieve all sizes of the Piccolo occluder based on bench testing. In contrast, bench testing demonstrates that the 4F TorqVue LP catheter cannot be used to fully recapture the Piccolo occluder consistently.
- For devices in the pulmonary circulation position a retrieval sheath into the MPA and minimize the number of times the tricuspid valve is crossed (Fig. 2). If the sheath cannot be safely advanced or causes hemodynamic compromise (hypotension, bradycardia or desaturation), retract the sheath into the right ventricle or into the right atrium while maintaining guidewire position in the descending aorta via the PDA. If needed, maintain a buddy guidewire down the descending aorta via the PDA to stabilize the sheath position within the MPA.
- Depending on the location of the device, select a 4F diagnostic catheter (as an alternative to the traditional snare catheter) to be used for snaring with a curve and characteristics suitable for reaching the vessel containing the device (such as JR2 for right pulmonary artery). Advance the catheter toward the device and grab the device with the snare.
- When snaring the device, it is not necessary to grab the pin/screw and it is acceptable to snare the device wherever it can be grabbed because it is soft and can easily be pulled into the retrieval sheath. Advance the snare beyond the device and spin to entangle the device. It sometimes may be necessary to try different snare types to be able to snare the device successfully. A typical location for snaring is in region between the retention disc and the central waist.
- Once the device is grasped with the snare, recapture the device into the retrieval sheath and pull the device through the sheath under fluoroscopic guidance and externalize.
- If the infant does not tolerate having the retrieval sheath across the heart in the pulmonary artery and becomes unstable there are two options:
- Bring back the retrieval sheath into the right atrium while keeping the diagnostic catheter in the MPA to permit snaring. Once snaring is accomplished, the retrieval sheath may be brought forward over the diagnostic catheter to the MPA to permit recapture of the device into the retrieval sheath.
- If the retrieval sheath cannot be advanced easily into the pulmonary artery, a snared device in the pulmonary circulation can gently be brought into the right ventricle and recaptured into the retrieval sheath in the RV or if necessary, brought back further into the RA before recapturing into the sheath in the RA or IVC. Extreme caution must be taken to avoid disruption of the pulmonic or tricuspid valves.
- In the event the retrieval procedure is prolonged, the procedure is not well tolerated, and the device does not appear to be causing any immediate danger to the infant (e.g., angiography shows good flow around the device without causing pulmonary branch obstruction), it may be reasonable to consider abandoning any further attempts to retrieve the device and postpone to a later time or consult with a surgeon for surgical retrieval of the device.
- In cases where the device is retrieved without difficulty, consideration may be given to implantation of a different device. Sometimes a larger device may not be suitable and referral for surgical ligation may be reasonable. Following instrumentation of the ductus, spontaneous closure may sometimes occur, in which case, no further intervention is required.

### Percutaneous Retrieval of an Embolized PDA Occluder from the Systemic Circulation

For device embolization into the systemic circulation prompt recognition and treatment is warranted. This is because device embolization into the systemic circulation is unlikely to be tolerated by small infants (< 2 kg) and will result in acute symptoms characterized by abdominal distention to full blown necrotizing enterocolitis, decreased lower extremity pulse and perfusion, oliguria and renal failure. Device embolization into the aorta requires emergency intervention and potentially may be amenable to transcatheter retrieval depending on whether a retrieval sheath may be introduced safely across the ductus into the aorta, or from the carotid artery into the aorta.

The following additional management considerations are recommended for device embolization into the descending aorta:

- For a device embolization in the systemic circulation assess the feasibility of transcatheter retrieval via the ductus or carotid artery; or consult with a surgeon for retrieval via a surgical approach.
- If the device embolizes into the descending aorta during the procedure, a long sheath may be introduced via the existing femoral venous access site, across the ductus to snare and retrieve the device in a similar fashion to the techniques used in the pulmonary circulation [22, 23].
- For a device that embolizes post-procedure two options exist for transcatheter retrieval depending on whether the ductus is patent. If the ductus is patent, then a long sheath may be introduced via the femoral vein, across the ductus to snare and retrieve the device. If the ductus has closed, then a carotid approach can be less harmful than a femoral arterial approach for infants < 2 kg. A long sheath is not necessary from the carotid artery, as long as the tip of the sheath is beyond the takeoff of the carotid from the aortic arch.
- For a device that embolizes post-procedure and is not detected until after weeks to months post implant it may not be feasible to retrieve the device with a transcatheter approach due to tissue ingrowth and device endothelialization. If the patient is asymptomatic, then observation alone is all that may be needed until such time that the patient is large enough for a safe surgical retrieval if necessary.
- If there are challenges maintaining a retrieval sheath across the ductus during attempts to recapture the device in the descending aorta, the sheath may be retracted into the MPA, RV, or RA. However, once the device is snared it should not be retrieved across the ductus unguarded and the retrieval sheath must be re-advanced across the ductus into the descending aorta prior to device retrieval.

## Device Protrusion and Aortic and Pulmonary Artery Obstruction

Device protrusion is a known complication following transcatheter PDA closure. The need for intervention depends on the degree of protrusion and resultant aortic or pulmonary artery obstruction [24, 25]. The potential for device protrusion resulting in vascular obstruction is greatest in smaller infants. Therefore, device sizing and placement in infants ≤ 2 kg is chosen to achieve a completely intraductal position with the goal of minimizing the potential for device protrusion.

### Incidence, Mechanism, and Clinical Presentation

The incidence of clinically significant aortic or left pulmonary artery (LPA) obstruction with the Piccolo occluder in infants ≤ 2 kg is 2%. This was based on two cases of aortic obstruction and no cases of LPA obstruction among 100 infants enrolled in the premarket trial [10]. In comparison, the reported incidence of major aortic or LPA obstruction based on a review of recent literature was 0.9% (range 0 to 3.8%) and 1.8% (range 0 to 4.1%), respectively, [13–18]. Most cases of aortic or LPA protrusion may be detected at the time of device implant, allowing for immediate corrective intervention, but sometimes the device may appear to be in ideal position at the time of implant and post implant the device might migrate and cause aortic or LPA obstruction. The incidence of post-procedure device migration in the premarket trial was 1% [10]. The time course for the development of post-procedure device obstruction is usually within 24 h, but sometimes may occur more gradually, and an obstruction may not be detected until several days to weeks post implant [24–26].

Table 5 outlines potential mechanisms for device protrusion resulting in aortic and LPA obstruction. The most common cause for device protrusion is device malposition. This may occur whenever there are difficulties deploying the device within an intraductal position in small infants, or secondary to using a larger or longer device than recommended in the IFU [19]. The stiffness of the delivery system relative to patient size also contributes to improper device positioning, especially in those < 1 kg. Relying exclusively on an esophageal temperature probe as a landmark to identify the aortic end of the ductus can also lead to improper device positioning within the ductus. Other mechanisms for device protrusion include ductal vasoconstriction on the pulmonic end causing the device to be pushed out the aortic end, and inadvertently pulling the device toward the LPA following device deployment. Inadvertent positioning of the device such that the superior edge of the aortic disc protrudes slightly into the aorta [27] may not be recognized at the time of implant but may subsequently develop into aortic obstruction as the ductus undergoes vasoconstriction and the device may be extruded further by blood flow outside the ductus toward the descending aorta. Lastly, device protrusion may be caused by device migration following release from the delivery wire, or from post-procedure device lengthening due to ductal vasoconstriction [25].

**Table 5:** Mechanisms for Device Protrusion

Device Size	Implanted device is too large for the encountered anatomy
Delivery System	Anterior tension on the device by delivery wire. Delivery catheter preventing device to stay co-axial along the length of the duct
Inadequate Imaging	Inability to adequately visualize location of aortic disc relative to aorta. Relying exclusively on temperature probe to identify aortic end of the ductus
Ductal Vasoconstriction	Post-procedure ductal vasoconstriction on pulmonic end causes device to be pushed out of aortic end; or vasoconstriction causing device lengthening
Device Malposition	Device positioned in small infant (≤ 2 kg) with one or both discs in an extraductal position. Difficulty in device positioning due to ductal distortion by delivery catheter
Operator related	Device pulled inadvertently into left pulmonary artery during or after deployment. Prolonged time interval between device placement and release
Migration	Device migrates following device release
Duct Orientation	Acute angulation of the ductus relative to the descending aorta resulting in more exposure of the superior edge of the aortic disc into the aortic lumen

Aortic obstruction following transcatheter PDA closure may sometimes occur in the absence of device protrusion. This may occur whenever there is an unrecognized juxtaductal aortic coarctation where blood flows across the coarctation and passes through the aortic ampulla region of the ductus [26, 28]. Once the ductus is closed with the device, flow through the aortic ampulla is no longer possible, and the aortic coarctation is unmasked and becomes clinically significant. In the premarket trial there was one infant (1%) with what was believed to be a mild juxtaductal aortic coarctation that became severe following transcatheter PDA closure unrelated to device positioning. This was successfully managed using an intravascular stent implanted in the aortic segment with the coarctation three days post implant using a carotid cutdown approach. Ultimately, this infant underwent successful surgical coarctation repair at 16 months of age.

Mild protrusion of the device into the LPA or aorta appears to be well tolerated in most infants > 1 kg, and after a period of several months to one year of monitoring the obstruction typically resolves without requiring any intervention as the infant grows [23]. However, in some cases involving small infants (≤ 2 kg) the degree of aortic and LPA obstruction may become clinically significant and result in symptoms requiring intervention [25]. The clinical presentation for aortic obstruction consists of a differential blood pressure between the upper and lower extremities (mean Doppler gradient ≥ 20 mmHg at the aortic isthmus by TTE), diminished lower extremity pulses and in some cases, there may be decreased renal function, necrotizing enterocolitis and bowel ischemia, worsening respiratory status, and even acidosis depending on the degree of obstruction. Acute decompensation due to aortic obstruction is more commonly seen in infants < 1 kg. The clinical presentation for LPA obstruction typically does not have any associated symptoms in those > 1 kg, but there is an elevated LPA gradient (peak instantaneous Doppler gradient ≥ 35 mmHg or peak velocity > 3 m/s). With a mismatch between left and right lung perfusion there may be an impact on left lung development. To ensure that aortic or LPA obstruction is not missed, it is important to monitor all infants ≤ 2 kg post implant for changes in aortic and pulmonary blood flow. Infants weighing < 1 kg may acutely decompensate, manifesting lower oxygen saturations, right ventricular hypertension and/or right-to-left shunting across the foramen ovale with obstruction to the LPA. This requires emergent retrieval of the device using techniques described previously.

### Prevention

Device protrusion is best prevented in infants ≤ 2 kg by selecting a device size that allows for easy intraductal device positioning. Both TTE and angiography should be used to guide device placement and confirm absence of aortic and LPA protrusion prior to releasing the device from the delivery wire.

**Fig. 3:**
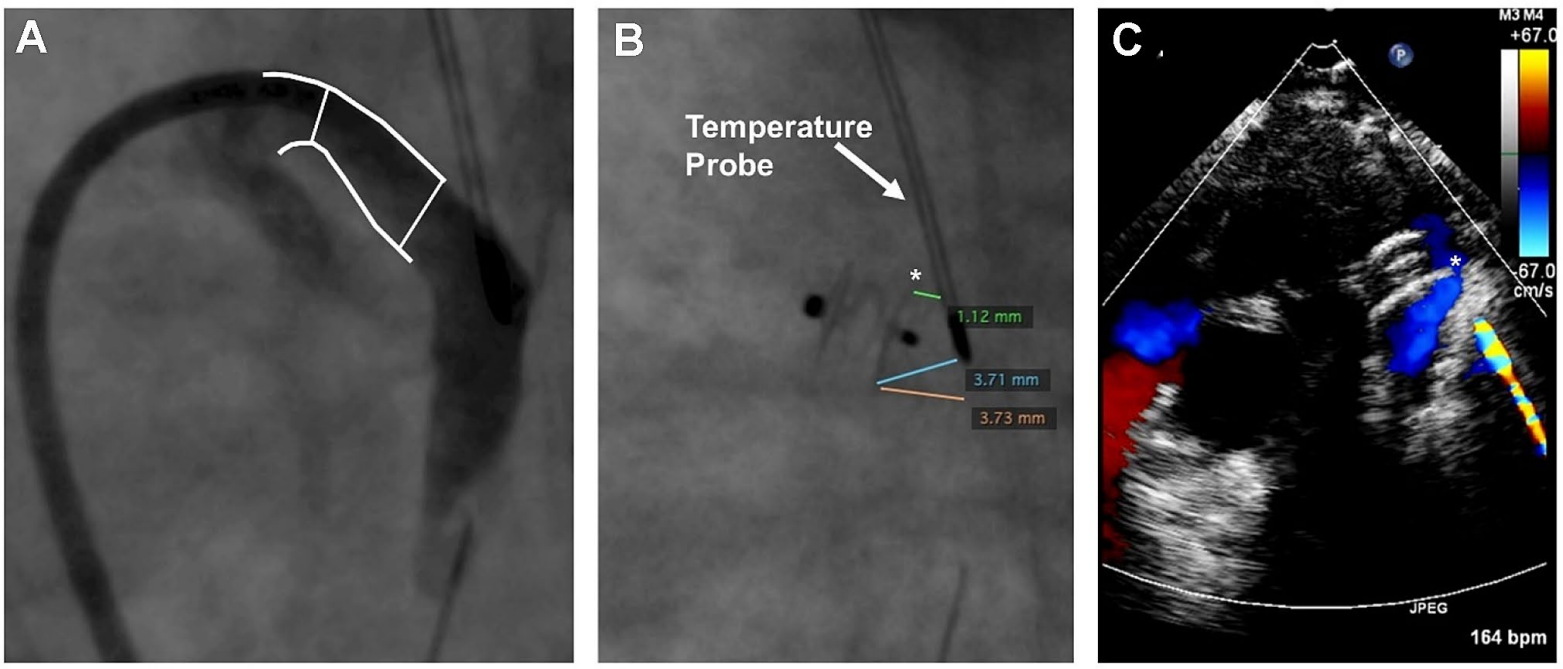
F-type PDA angiogram in 720-g infant (**A**) followed by closure using the Amplatzer Piccolo occluder device position relative to temperature probe (**B**) followed by aortic coarctation seen six hours post implant on echocardiogram (**C**). Asterisk (*) marks superior edge of aortic disc protruding into aorta

- In small infants (≤ 2 kg) the length of the occluder is chosen to be shorter than the length of the PDA to achieve intraductal positioning with a preference to use the 2 mm length device in infants ≤ 1 kg. If a decision is made to use a 4 mm length device, it is important to ensure that the PDA length is at least 12 mm and that the entire device is implanted intra-ductal. If the entire device can be implanted intra-ductal, there may be a scenario where a smaller diameter device with more length (3 mm × 4 mm) may fit the anatomy better compared to a larger diameter device with a shorter length (4 mm × 2 mm).
- Placement of an esophageal temperature probe pre-procedure may serve as a useful landmark of the aortic isthmus in small infants (≤ 2 kg). However, it is important to recognize that the exact position of the temperature probe relative to the anterior aortic wall at the level of the ductus should be interpreted with caution since it varies across patients and depends on image angulation. Use angiography to determine the position of the temperature probe relative to the ductal ampulla and if needed consider optimizing the imaging projection angulation to achieve better alignment of the temperature probe.
- In infants with an upper extremity central line in the superior vena cava (SVC), the tip of the central line may mark the pulmonary artery end of the PDA and serve as a useful landmark for device positioning. However, similar to the temperature probe the exact position of the tip of the central line relative to the PDA should be interpreted with caution.
- For small infants (≤ 2 kg), it may be necessary to deploy the aortic disc within the PDA to achieve an intraductal position. Deployment of the aortic disc in the descending aorta followed by retraction into the ductus may not always result in the entire disc entering the ductus. In particular, the superior edge of the aortic disc may protrude into the aorta whenever the ductus has an acute angle into the descending aorta and device positioning favors the aortic end of the ductus (Fig. 3) [27]. The device ideally should be deployed to achieve a central position along the ductus aiming to place the proximal disc within the pulmonary ampulla while ensuring there is no LPA protrusion.
- For small infants (≤ 2 kg), it may be necessary to push the device forward while retracting the catheter to fully pack the device within the duct and achieve an intraductal position and avoid protrusion into the LPA.
- A “football” shaped disc may be indicative of a device diameter that may be too large. This disc configuration could eventually flatten out and bring the disc to lie in an extraductal location. Therefore, if the disc does not flatten within the PDA, then changing to a one size smaller device may be necessary.
- Following device deployment and prior to releasing the device from the delivery wire, it is important to rely on intra-procedural TTE and fluoroscopy/angiography to ensure the device is in a co-axial position and proper orientation (10 o’clock) without obstructing the aorta or LPA. If there is evidence of aortic or LPA protrusion, recapture the entire device and reposition to achieve an intraductal position. If the device cannot be repositioned successfully, the device may be too large, and consideration should be given to either using a smaller device or pursuing an alternative approach for treating the PDA. Recapturing only the proximal portion of the device and redeploying using a “packing-type” approach can be effective for LPA protrusion, but less likely to work for an aortic obstruction where complete device recapture is recommended.
- TTE may be used to assess the Doppler velocity and waveform pattern to rule out an aortic or LPA protrusion. A Doppler velocity greater than 2.5 m/s in combination with an obstructive flow pattern and two-dimensional color Doppler imaging showing possible protrusion should not be ignored and warrants repositioning the device if can be done safely. Following PDA closure, the Doppler velocity in the descending aorta typically decreases, while the Doppler velocity in the LPA increases slightly, and in most cases the velocity remains below 2 m/s [29]. Intra-procedural Doppler velocity alone may not be a reliable indicator to declare an increased or decreased risk for subsequent obstruction and must be used in context with other imaging modalities noted herein.
- Once the device has been deployed and prior to release, a delicate balance exists between using too much time performing a TTE to assess device positioning and absence of aortic obstruction prior to release from the delivery wire versus causing proximal device migration while attached to the delivery wire. Prolonged duration may cause the device getting pulled anterior by the tension of the delivery system and the device may no longer be properly positioned by the time the device is released.
- Assess femoral pulses and lower extremity pulse oximetry tracing before and after device deployment to rule out aortic obstruction. Comparison of blood pressure cuff readings between the upper and lower extremities can also be helpful to rule out aortic obstruction.
- Angiograms performed with the device attached to the delivery wire through the sidearm of the delivery catheter can rule out LPA obstruction with the aortic end visualized on levophase. Useful projections include 90° lateral and 30° LAO with 10°-15° cranial angulation.
- Following device release from the delivery wire there is a potential for the device to shift in position and result in aortic or LPA protrusion. Therefore, it is important prior to leaving the cardiac catheterization laboratory to perform a thorough imaging assessment to ensure there is no device protrusion.
- If there is device-related LPA or aortic arch obstruction noted before transport of the child from the catheterization laboratory, considerations to re-intervention should be given.

### Management

**Fig. 4:**
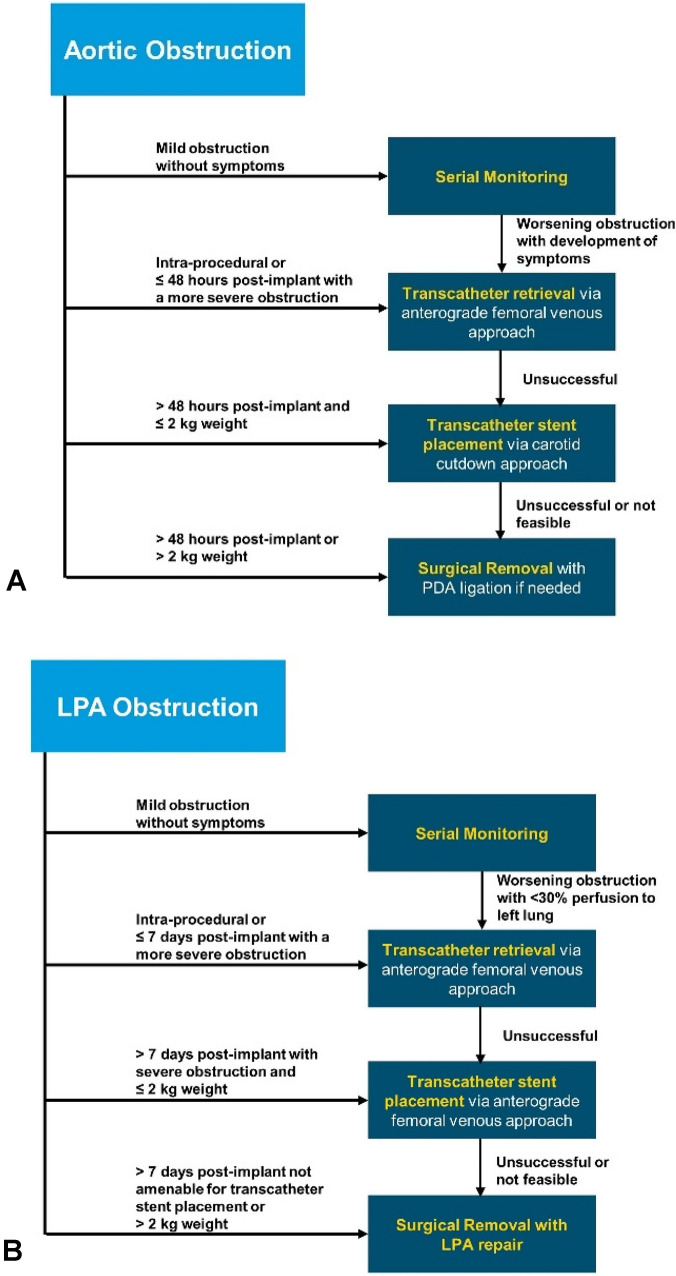
Algorithm to manage aortic obstruction (**A**) and LPA obstruction (**B**)

Whenever a device protrusion is identified either intraprocedurally or post implant, the degree of aortic or LPA protrusion and associated clinical status should be carefully assessed. Depending on the findings, urgent intervention may be required, or it may be acceptable to not intervene and continue monitoring. A management algorithm for device protrusion is outlined in Fig. 4 along with the following management guidelines.

**Fig. 5:**
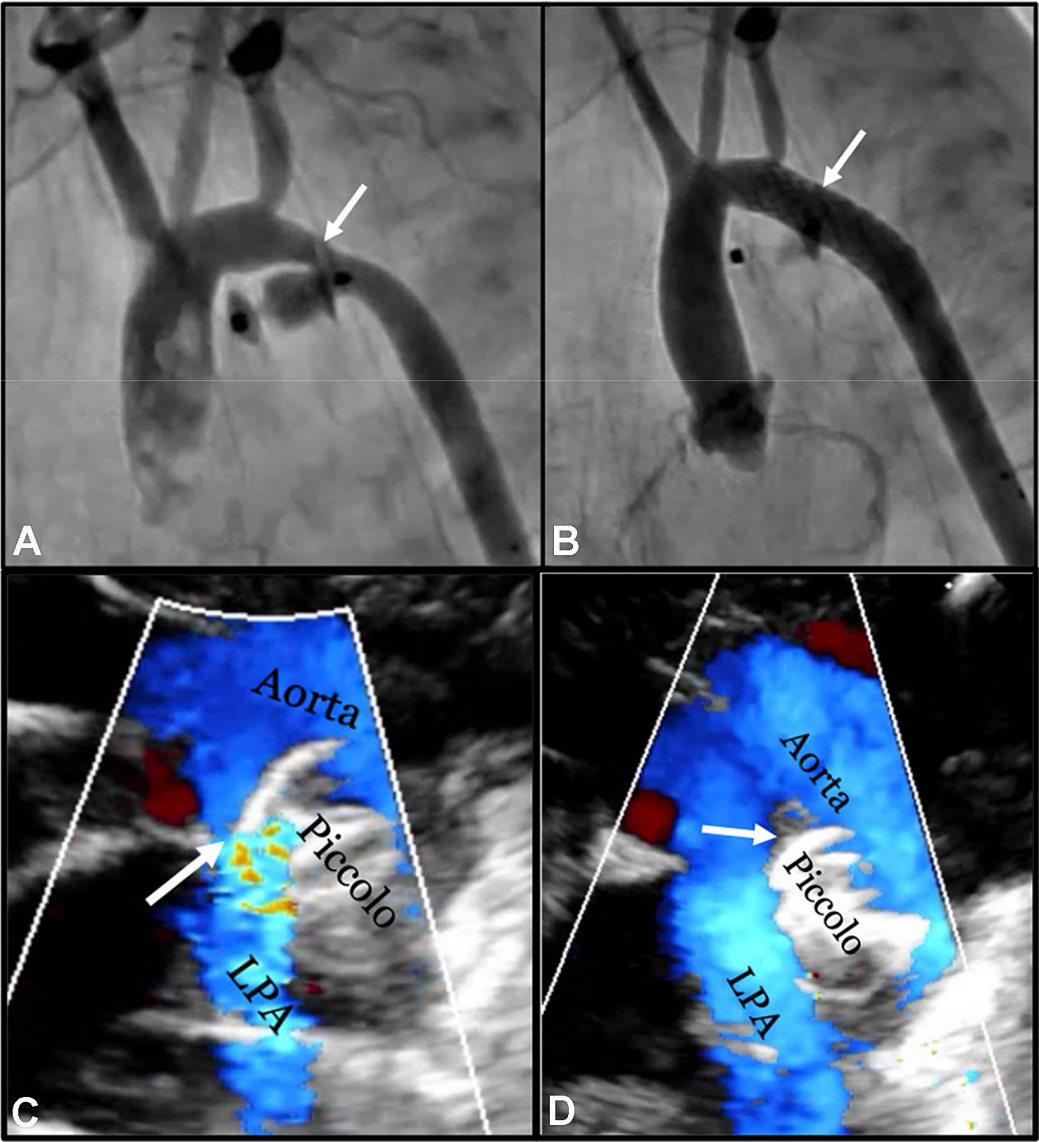
Device Protrusion Causing Aortic or Left Pulmonary Artery Obstruction. **A** Extraductal implantation with distal disc projecting into the aorta leading to aortic arch obstruction (ARROW) in a 540-g infant. **B** Aortic arch obstruction in the infant (A) treated with stent implantation from a carotid approach with no residual stenosis (ARROW). **C** Extraductal implantation with proximal disc projecting into the LPA (ARROW) leading to LPA stenosis in an 800-g infant (echocardiographic parasternal ductal view). **D** Intraductal repositioning of the device (ARROW) in the infant (C) with disc no longer causing LPA stenosis

- Infants with evidence of aortic or LPA protrusion should be closely monitored for potential signs of vascular obstruction and treated promptly whenever there are signs of clinical compromise. Limb or intestinal ischemia, worsening respiratory status, hypoxia, decreasing urine output, or acidosis secondary to vascular obstruction caused by the device requires urgent intervention.
- Intervention may consist of a transcatheter device retrieval, transcatheter stent placement [28], or surgical intervention to remove the device and relieve the obstruction depending on the feasibility to perform safely (Fig. 5).
- When contemplating transcatheter retrieval of the device, femoral arterial access should not be utilized in small infants ≤ 2 kg to retrieve the device secondary to the risk of causing vascular injury with limb ischemia.
- Infants ≤ 1 kg with a significant aortic obstruction are at an increased risk for a fatality if not treated promptly. Larger infants with evidence on TTE of a significant aortic obstruction (Doppler velocity >2.5 m/s), but without symptoms and without a significant blood pressure differential between the upper and lower extremities (≥ 20 mmHg) may be expectantly managed with close follow-up.
- LPA obstruction (Fig. 5) without associated symptoms often may be managed conservatively without intervention. However, when lung perfusion scan demonstrates less than 30% flow to the left lung, intervention may be considered. There have been instances where LPA obstruction completely resolved by simply waiting for the infant to grow.
- Following retrieval of the device it may be reasonable to use another device to occlude the ductus whenever the ductus remains patent and the infant is clinically stable.
- It may be prudent to implant a stent from a carotid artery approach to treat aortic arch obstruction in infants < 1kg rather than trying to retrieve the device. Device retrieval in such small infants, especially 24-48 hours post procedure can result in significant hemodynamic compromise. Therefore, stent implantation to treat aortic obstruction may be a more prudent treatment option to stabilize these very small infants, with future reinterventions required when the child is older.

## Tricuspid Valve Regurgitation

### Incidence, Mechanism, and Clinical Presentation

Tricuspid valve regurgitation (TR) is a potential complication following transcatheter PDA closure that may occur whenever utilizing the anterograde transvenous approach for delivering the device. The incidence of TR among infants ≤ 2 kg was 5% in the premarket trial [10]. In comparison, the reported incidence of procedure -related TR based on a review of recent literature was 2.2% (range 0 to 4.1%) [13–18]. New onset or worsening of existing TR is most commonly detected intraprocedurally or immediately following the procedure.

We believe the most common cause of TR associated with this procedure is injury to the chordae of the septal leaflet of the tricuspid valve which occurs during catheter passage across the valve. This can occur secondary to a variety of etiologies:

- Initial passage of the catheter between the chordae rather than between the valve leaflets themselves.
- Mismatch between a guidewire and the internal lumen of a catheter resulting in chordae entrapment [17].
- A lengthy and challenging implant procedure, particularly involving device embolization with multiple crossings of the tricuspid valve with various catheters and sheaths.
- Retrieval of an embolized device unguarded (i.e., not within a retrieval sheath) through the tricuspid valve.

### Prevention

TR is best prevented by minimizing the number of times the tricuspid valve is crossed and ensuring that the tricuspid valve is always crossed centrally in an atraumatic fashion. Tips for accomplishing this include:

- Never advance a catheter over a guidewire across the tricuspid valve without imaging guidance if resistance is encountered, or if there is significant mismatch between the catheter lumen and the guidewire diameter.
- Two approaches have been used for crossing the tricuspid valve:
- The first approach uses a 4 French angled glide catheter (Radifocus™ Glidecath™ Non-Taper ANGLE RF * ZV9410GA - 4Fr x 65cm x .038", CG415, Terumo, Japan) that is advanced over a floppy atraumatic tip 0.035-inch wire. Once in the right atrium, the catheter is pointed toward the tricuspid valve and the wire advanced into the RV. The wire and the guide catheter are gradually advanced through the heart while taking advantage of the angled catheter tip to guide the wire into the RV initially followed by the pulmonary outflow tract. The guidewire can then be advanced across the PDA down the descending aorta. Subsequently, the delivery catheter can be passed directly over this 0.035” wire with insignificant wire-catheter mismatch (Amplatzer TorqVue LP Catheter, 4Fr x 80cm x 0.046”, Abbott, Plymouth, MN, USA).
- The second approach uses a 4 French balloon end-hole catheter advanced to the mid-right atrium and directed and passed across the tricuspid valve with the assistance of a curved stiff end of an 0.018-inch guidewire that must remain within the catheter lumen. A 0.014-inch floppy tipped coronary guidewire is then advanced out the pulmonary artery across the PDA into the descending aorta. With this approach there is significant mismatch between the 0.014” guidewire and the delivery catheter lumen and it is strongly recommended that this be addressed by placing a 0.21” microcatheter coaxially within the delivery catheter before passing it over the guidewire.
- Retrieval of an embolized device from the pulmonary artery should be performed through a long sheath to minimize the potential for injuring the tricuspid valve. If a long sheath cannot be utilized during retrieval, an unguarded device may be gently pulled through the right heart under imaging guidance. Use of TTE during this process may permit retrieval through the valve while limiting the formation of TR. If resistance is encountered, further attempts to retrieve the device should be abandoned and an alternative interventional technique or a surgical approach for device retrieval may be needed.

### Management

Mild to moderate TR appears to be well tolerated in most infants and can be managed without further intervention. Severe TR may be associated with worsening heart failure and respiratory status and in the presence of a patent foramen ovale or an atrial septal defect may result in right-to-left shunting resulting in systemic arterial desaturation. In larger infants, surgical intervention may be possible. However, management of severe TR in very small premature infants is currently limited to medical therapy as the risks for surgical intervention are too great. Therefore, severe TR in this population may be associated with poor clinical outcomes.

## Cardiovascular Injury

### Incidence, Mechanism, and Clinical Presentation

Cardiovascular injury is a known potential complication as a result of instrumentation during transcatheter PDA closure. Cardiovascular injuries may range from minor vascular access site injuries to more serious complications such as inferior vena cava disruption and cardiac perforation [42]. In the premarket trial there was one (1%) non-serious vascular access complication in infants ≤ 2 kg. There were no other serious cases of vascular injury or cardiac perforation. In comparison, the reported incidence of serious cardiovascular injuries based on a review of recent literature was 1.3% (range 0 to 8.3%) corresponding to three cases of cardiac perforation that occurred during guidewire and catheter manipulation through the right heart [13–18]. The cardiac perforation in two of these cases resulted in a procedure-related mortality, while in the third case, the infant was rescued with surgical repair of a right atrial perforation.

The most common cause for cardiac perforation or vascular injury is due to technical difficulty in advancing a guidewire or catheter through the vasculature or heart, and utilizing excessive force when resistance is encountered. Vascular access site injuries are characterized by bleeding with a groin hematoma, femoral artery thrombosis, and/or loss of peripheral pulses. Inferior vena cava or ductal disruption is characterized by major internal bleeding with hypotension necessitating emergency intervention. Cardiac perforation is characterized by a pericardial effusion or cardiac tamponade requiring emergency intervention.

### Prevention

- Do not deliver the device in small infants (≤ 2 kg) using the retrograde approach as small infants are at an increased risk for arterial injury. In small infants, it is recommended to deliver the device using an anterograde transvenous approach only. Arterial access in this population should be reserved for emergencies only and may be best accomplished using a surgical cutdown.
- Utilization of a vascular ultrasound to guide femoral vein access may further decrease the risk of inadvertently accessing the femoral artery [30–32]. Once access is gained with a guidewire to the femoral vein, confirm guidewire position with fluoroscopic imaging prior to insertion of an introducer.
- Whenever advancing guidewires and catheters through the vasculature rely on fluoroscopic guidance to prevent damage to the vessels and cardiac tissue. Do not advance a catheter over a guidewire without image guidance if resistance is encountered. Advancing the catheter under such circumstances has the potential to result in cardiovascular injury.
- If there is suspicion for cardiac perforation, immediate TTE to identify a pericardial effusion and prompt treatment can be lifesaving.

### Management

- Vascular access site injury with formation of a hematoma may be managed conservatively as long as the hematoma is not expanding. Femoral artery thrombosis may be treated with either short-term intravenous anticoagulation (unfractionated heparin) or aspirin if there are no systemic contraindications.
- Cardiac perforation with formation of a pericardial effusion requires emergency intervention to drain the pericardial effusion and repair the site of perforation. Pericardiocentesis under TTE guidance and/or cardiac surgery may be needed to prevent a fatality particularly in the setting of cardiac tamponade. Pericardiocentesis may be accomplished using a 4 French micro-introducer kit (Galt Medical, REF#KIT-002–34) to access the pericardial space followed by drain placement (Cook Pediatric Pericardiocentesis Set, 5 Fr; 30 cm; 0.028; REF#G05251).
- Utilization of a clear, plastic drape will allow visualization of the infant under the drape at all times. This may potentially help identify hemoperitoneum from accidental perforation sooner allowing for prompt intervention.
- In case of a large perforation, or vascular injury with massive hemorrhage, consider auto-transfusion by drawing up the bleeding from the pericardium, pleura or peritoneum until the bleeding site can be identified and effectively controlled or surgically repaired.
- Call for surgical backup immediately in case of cardiac or vascular perforation.

## Residual Shunt and Hemolysis

A residual shunt following transcatheter PDA closure occurs with an incidence < 1% based on the premarket clinical trial [10]. A residual shunt is most commonly due to a device malposition but may also be a result of device migration. In the premarket trial there were two cases among 200 implants (1%) of post-procedural device migration that occurred within 24 h post-procedure and were associated with a residual shunt [10]. In both cases a transcatheter retrieval was performed successfully. Another but less common cause for a residual shunt is congenital thrombocytopenia [10]. In the presence of a residual shunt hemolysis leading to hyperbilirubinemia may occur due to high flow through a narrow channel [7, 33]. If the residual shunt does not resolve, intervention may be required to seal the shunt or to retrieve the device and close the PDA. If the device position is proper and residual shunting is due to thrombocytopenia, it may be reasonable to wait for the residual shunt to resolve while providing supportive care and stringent monitoring. However, if residual shunting is due to a device malposition and does not appear to resolve, it may be best to proceed with an intervention.

## Contrast Induced Nephropahty

To minimize the potential for contrast induced nephropathy either a minimal volume of contrast (2 to 4 mL) or no contrast is utilized in premature infants with pre-existing renal dysfunction [15–18]. In the premarket trial the average amount of contrast utilized was 2.5 ± 1.7 mL/kg with no infants experiencing contrast induced nephropathy [10]. It has been shown that the implant procedure can be performed safely without the use of contrast, but both fluoroscopic and TTE guidance are important to ensure procedural success [7, 33].

## Summary

Early clinical experience with transcatheter PDA closure in ELBW infants using the Amplatzer Piccolo Occluder shows promising clinical outcomes [10, 34–41, 42, 43]. Although the incidence of periprocedural complications is low, the potential for these complications to result in major morbidity and/or mortality is significant. It is, therefore, imperative that implanters of the Piccolo Occluder are aware of all potential complications and develop a strategy to prevent, promptly recognize, and manage them. We believe that structured education and guidelines for aspiring implanters coupled with experienced onsite physician proctoring for initial cases and continued expert field support are crucial in maintaining the low incidence of complications observed in the premarket clinical trial. While this emerging technology clearly has an important role to play in the management of these tiny infants, further study and device/delivery system improvements should be pursued to maximize the ultimate benefit to this vulnerable population.

## References

[1] Sathanandam S, Agrawal H, Chilakala S (2019). Can transcatheter PDA closure be performed in neonates ≤1000 grams? The Memphis experience. Congenit Heart Dis.

[2] Almeida-Jones M, Tang NY, Reddy A, Zahn E (2019). Overview of transcatheter patent ductus arteriosus closure in preterm infants. Congenit Heart Dis.

[3] Sathanandam S, Balduf K, Chilakala S (2019). Role of transcatheter patent ductus arteriosus closure in extremely low birth weight infants. Catheter Cardiovasc Interv.

[4] Fraisse A, Bautista C, Burmester M, Lane M, Singh Y (2020). Transcatheter closure of patent ductus arteriosus in infants with weight under 1,500 grams. Front Pediatr.

[5] Agrawal H, Waller BR, Surendan S, Sathanandam S (2019). New patent ductus arteriosus closure devices and techniques. Intervent Cardiol Clin.

[6] Bischoff AR, Jasani B, Sathanandam SK, Backes C, Weisz DE, McNamara PJ (2020). Percutaneous closure of patent ductus arteriosus in infants 1.5 kg or less: a meta-analysis. J Pediatr.

[7] Johnson JN, Sathanandam S, Naik R (2019). Echocardiographic guidance for transcatheter patent ductus arteriosus closure in extremely low birth weight infants. Congenit Heart Dis.

[8] Paudel G, Johnson JN, Philip R, Tailor N, Fahnhorst S, Briceno-Medina M, Stecchi N, Waller BR, Sathanandam S (2021). Echocardiographic versus Angiographic Measurement of the Patent Ductus Arteriosus in Extremely Low Birth Weight Infants and the Utility of Echo Guidance for Transcatheter Closure. J Am Soc Echocardiogr.

[9] Ranjit Philip B, Waller R, Agrawal V, Wright D, Arevalo A, Zurakowski D, Sathanandam S (2016). Morphologic characterization of the patent ductus arteriosus in the premature infant and the choice of transcatheter occlusion device. Catheter Cardiovasc Interv.

[10] Sathanandam SK, Gutfinger D, O'Brien L, Forbes TJ, Gillespie MJ, Berman DP, Armstrong AK (2020). Amplatzer Piccolo Occluder clinical trial for percutaneous closure of the patent ductus arteriosus in patients ≥ 700 grams. Cathet Cardiovasc Intervent.

[11] Vali P, Lakshminrusimha S, Pelech A, Underwood M, Ing F (2019). Patent ductus arteriosus in preterm infants: is early transcatheter closure a paradigm shift?. J Perinatol.

[12] Backes CH, Kennedy KF, Locke M, Cua CL, Ball MK, Fick TA, Rivera BK (2017). Transcatheter occlusion of the patent ductus arteriosus in 747 infants < 6 kg: insights from the NCDR IMPACT registry. JACC: Cardiovasc Intervent.

[13] Baspinar O, Sahin DA, Sulu A, Irdem A, Gokaslan G, Sivasli E, Kilinc M (2015). Transcatheter closure of patent ductus arteriosus in under 6 kg and premature infants. J Intervent Cardiol.

[14] Ogando A, Rodríguez IP, Asensio AR, Sánchez de la Blanca F, Ballesteros Tejerizo M, Sánchez Luna JM, Gil Jaurena C, López M, Zunzunegui JL, Martínez (2018). Surgical ligation versus percutaneous closure of patent ductus arteriosus in very low-weight preterm infants: which are the real benefits of the percutaneous approach?. Pediatr Cardiol.

[15] Morville P, Akhavi A (2017). Transcatheter closure of hemodynamic significant patent ductus arteriosus in 32 premature infants by amplatzer ductal occluder additional size-ADOIIAS. Catheter Cardiovasc Interv.

[16] Pamukcu O, Tuncay A, Narin N, Baykan A, Korkmaz L, Argun M, Ozyurt A, Sunkak S, Uzum K (2018). Patent ductus arteriosus closure in preterms less than 2 kg: surgery versus transcatheter. Int J Cardiol.

[17] Malekzadeh-Milani S, Akhavi A, Douchin S, Dauphin C, Chalard A, Mauran P, Bouvaist H, Bonnet D, Boudjemline Y (2020). Percutaneous closure of patent ductus arteriosus in premature infants: a French national survey. Catheter Cardiovasc Interv.

[18] Regan W, Benbrik N, Sharma S-R, Auriau J, Bouvaist H, Bautista C, Sirico D (2020). Improved ventilation in premature babies after transcatheter versus surgical closure of patent ductus arteriosus. Int J Cardiol.

[19] Instructions for Use of Amplatzer Piccolo Occluder. Abbott ARTEN600042307 B; 2020–07. Available on-line at https://manuals.sjm.com/?ct=professional.

[20] Batlivala SP, Glatz AC, Gillespie MJ, Dori Y, Rome JJ (2014). Ductal spasm during performance of transcatheter ductal occlusion. Catheter Cardiovasc Interv.

[21] Alkamali A, Alasrawi S (2018). Dealing with patent ductus arteriosus (PDA) spasm in the cath lab. J Cardiol Curr Res.

[22] Tan CA, Levi DS, Moore JW (2005). Embolization and transcatheter retrieval of coils and devices. Pediatr Cardiol.

[23] Alkhouli M, Sievert H, Rihal CS (2019). Device embolization in structural heart interventions: incidence, outcomes, and retrieval techniques. JACC Cardiovasc Intervent.

[24] Tomasulo CE, Gillespie MJ, Munson D, Demkin T, O'Byrne ML, Dori Y, Smith CL, Rome JJ, Glatz AC (2020). Incidence and fate of device-related left pulmonary artery stenosis and aortic coarctation in small infants undergoing transcatheter patent ductus arteriosus closure. Catheter Cardiovasc Interv.

[25] Chien Y-H, Wang H-H, Lin M-T, Lin H-C, Chun-Wei Lu, Chiu S-N, Mei-Hwan Wu, Wang J-K, Chen C-A (2020). Device deformation and left pulmonary artery obstruction after transcatheter patent ductus arteriosus closure in preterm infants. Int J Cardiol.

[26] Liu F, Hsiung M-C, Song H, Dian Ke, Tang H, Liu J (2013). Unexpected co-arctation of aorta detected by transesophageal echocardiography during patent ductus arteriosus ligation. Front Med.

[27] Masri S, El Rassi I, Arabi M, Tabbakh A, Bitar F (2015). Percutaneous closure of patent ductus arteriosus in children using amplatzer duct occluder II: relationship between PDA type and risk of device protrusion into the descending aorta. Cathet Cardiovasc Intervent.

[28] Dimas VV, Leonard SR, Guleserian KJ, Forbess JM, Zellers TM (2010). Stent implantation for coarctation of the aorta in a premature infant through carotid cutdown as a bridge to surgical correction. J Thorac Cardiovasc Surg.

[29] Markush D (2020). Fate of the left pulmonary artery and thoracic aorta after transcatheter patent ductus arteriosus closure in extremely low birthweight infants. Pediatr Cardiol.

[30] Alexander J, Yohannan T, Abutineh I (2016). Ultrasound-guided femoral arterial access in pediatric cardiac catheterizations: A prospective evaluation of the prevalence, risk factors, and mechanism for acute loss of arterial pulse. Catheter Cardiovasc Interv.

[31] Tadphale S, Yohannan T, Kauffmann T (2020). Accessing femoral arteries less than 3 mm in diameter is associated with increased incidence of loss of pulse following cardiac catheterization in infants. Pediatr Cardiol.

[32] Tadphale SD, Zurakowski D, Bird LE (2020). Construction of femoral vessel nomograms for planning cardiac interventional procedures in children 0–4 Years Old. Pediatr Cardiol.

[33] Pan X, Hijazi ZM, Sievert H, Pan X, Hijazi ZM, Sievert H (2020). Echocardiography-Guided Interventional Patent Ductus Arteriosus Closure by Femoral Vein Approach”. Percutaneous and Non-fluoroscopical (PAN) Procedure for Structural Heart Disease.

[34] Philip R, Waller BR, Chilakala S (2020). Hemodynamic and clinical consequences of early versus delayed closure of patent ductus arteriosus in extremely low birth weight infants. J Perinatol.

[35] Philip R, Lamba V, Talati A, Sathanandam S (2020). Pulmonary hypertension with prolonged patency of the ductus arteriosus in preterm infants. Children.

[36] Sathanandam S, Whiting S, Cunningham J (2019). Practice variation in the management of patent ductus arteriosus in extremely low birth weight infants in the United States: Survey results among cardiologists and neonatologists. Congenit Heart Dis.

[37] Philip R, Towbin JA, Sathanandam S (2019). Effect of patent ductus arteriosus on the heart in preterm infants. Congenit Heart Dis.

[38] Philip R, Nathaniel Johnson J, Naik R (2019). Effect of patent ductus arteriosus on pulmonary vascular disease. Congenit Heart Dis.

[39] Apalodimas L, Waller BR, Philip R (2019). A comprehensive program for preterm infants with patent ductus arteriosus. Congenit Heart Dis.

[40] Willis A, Pereiras L, Head T (2019). Transport of extremely low birth weight neonates for persistent ductus arteriosus closure in the catheterization lab. Congenit Heart Dis.

[41] Sathanandam S, Gianinni A, Sefton E (2019). Live broadcast of transcatheter PDA closure in a 700 grams ELBW infant during the International PDA Symposium. Congenit Heart Dis.

[42] Taylor R, Forbes MJ, Kobayashi D (2020). Transcatheter closure of patent ductus arteriosus in a tiniest baby–510 grams. Prog Pediatr Cardiol.

[43] Philip R, Tailor N, Johnson JN, Apalodimas L, Cunningham J, Hoy J, Waller III BR, Sathanandam S (2021). Single-Center Experience of 100 Consecutive Percutaneous Patent Ductus Arteriosus Closures in Infants ≤1000 Grams. Circ. Cardiovasc. Interv..

